# Association between hypomania and self-harm behaviors in Chinese children and adolescents with depressive symptoms

**DOI:** 10.3389/fpsyt.2022.870290

**Published:** 2022-08-24

**Authors:** Die Fang, Yusan Che, Hailiang Ran, Ahouanse Roland Donald, Sifan Wang, Junwei Peng, Lin Chen, Chuanzhi Xu, Yuanyuan Xiao

**Affiliations:** Department of Epidemiology and Health Statistics, School of Public Health, Kunming Medical University, Kunming, China

**Keywords:** hypomania, self-harm, children and adolescents, depressive symptoms, association

## Abstract

Bipolar disorder (BD) is associated with a higher risk of self-harm (SH) when compared with depression. Therefore, it is reasonable to suspect that the state of mania or hypomania may independently contribute to increased SH risk. However, for hypomania, its association with SH remains less known. We intend to investigate this hypothesis in a large sample of Chinese children and adolescents with depressive symptoms. Based on a two-stage simple random cluster sampling method with probability proportionate to sample size (PPS) design, a total of 4,858 children and adolescents aged between 10 and 17 years were surveyed in southwestern China, Yunnan Province, by using self-administered questionnaires. Among them, 1,577 respondents with depressive symptoms were screened out and included in the final analysis. Descriptive statistics were calculated to illustrate the major characteristics of the study subjects. Multivariate logistic regression models were fitted to evaluate the adjusted association between hypomanic symptoms and SH. The prevalence of SH in children and adolescents with depressive symptoms was 63.92% (95% CI: 58.70–69.00%). The two hypomanic factors, which measure “active/elated” (factor I) and “risk-taking/irritable” (factor II), were significantly and discordantly associated with SH: after adjustment, every one-point increase in factor I and factor II scores was associated with 0.94-fold (95% CI: 0.91–0.97) and 1.25-fold (95% CI: 1.15–1.36) of odds ratio (OR) in SH prevalence. Further analyses based on quartiles of the two factors revealed a more prominent dose–response relationship between factor II and SH prevalence, SH repetition, and SH severity. The results of this study may suggest that, for hypomanic children and adolescents, individuals with elevated factor II score are probably of greater urgency for SH intervention. Major limitations of this study include inability of causal inference, risk of information bias, and limited results extrapolation.

## Introduction

Self-harm (SH) refers to act of intentionally harming one's body or tissue with or without the intention of suicide (1). Compared to other age groups, young people are observed a higher risk of SH: it has been estimated that the prevalence of SH among adolescents reached 17.2% (2). A previously published meta-analysis has also found that SH behaviors were common among the Chinese adolescents, with a pooled SH prevalence of 22.37% (3). Prospective studies have observed that people who had repeated SH behaviors were four times more likely to report suicidal thoughts and behaviors in the next year (4). Considering the intimate relationship between SH and suicide, the intervention of SH behavior can be effective in proactively preventing suicide.

Depression is one of the most significant risk factors of SH in adolescents (5). Nevertheless, compared with depression, bipolar disorder (BD) is associated with an even higher risk of SH: a longitudinal study in the United States reported that the lifetime SH prevalence of BD was about three times that of depression (37–13%) (6). BD is a mood disorder characterized by periods of depression and periods of abnormally elevated happiness. If the elevated mood is severe, it is called mania; if it is less severe, it is called hypomania (7). BD with hypomania is usually misdiagnosed as depression (8), especially in children and adolescents who usually presented mild or unclear symptoms (9). Therefore, studies that reported increased SH risk for BD generally included maniac subjects, and whether the state of hypomania also independently contributes to SH risk remains less known.

**Figure 1:**
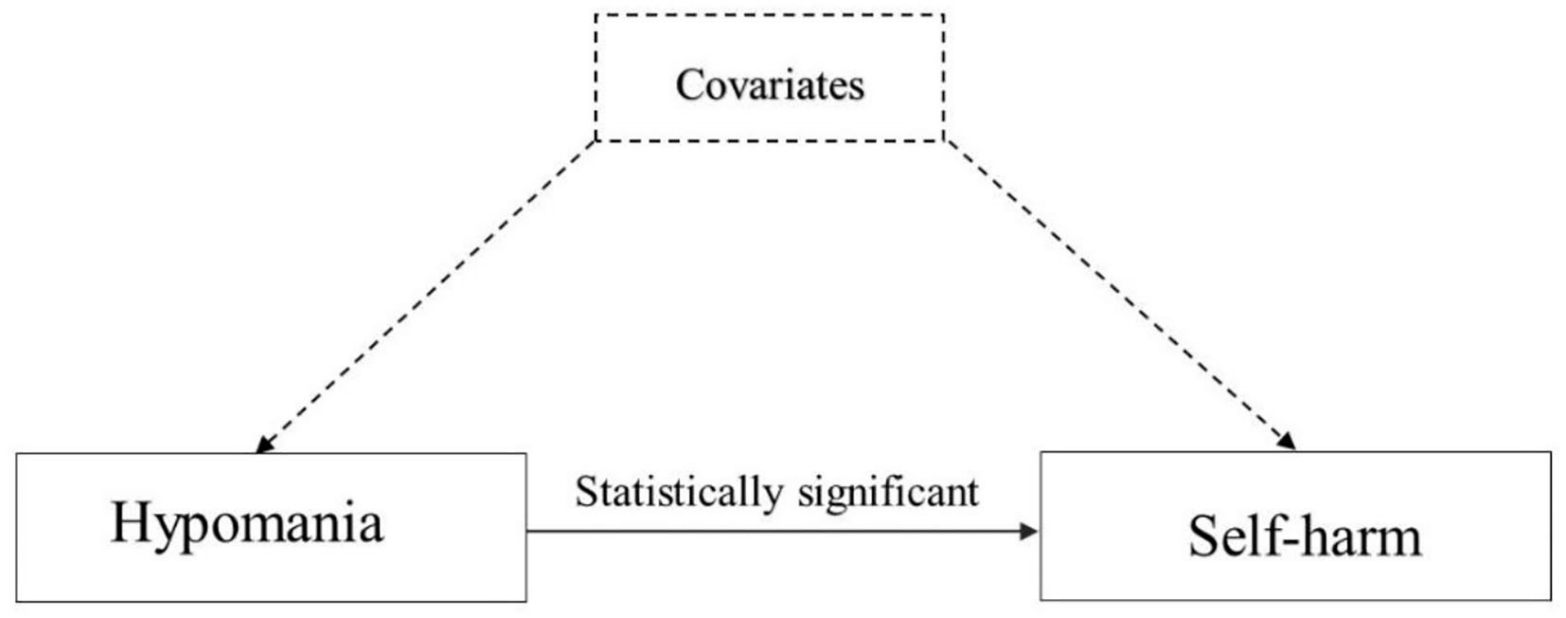
The hypothesized association between hypomania and SH.

The major aim of this study was to explore the independent association between hypomania and SH in the Chinese children and adolescents. As hypomania screening results are more accurate in populations with mood disorders, especially depression, we discussed this association only in teenagers who presented depressive symptoms. We put forward the following two major hypotheses: after controlling for major covariates, (1) hypomania is significantly and independently associated with SH (Figure 1); and (2) the two hypomanic factors show significant but discordant associations with SH.

## Materials and methods

### Study design

A cross-sectional survey was conducted in Kaiyuan City, Honghe Prefecture, Yunnan Province, China, from 19 October 2020 to 3 November 2020. A multistage simple random cluster sampling method with probability proportionate to sample size (PPS) design was used: in the first stage, 19 schools (eight primary schools, nine junior high schools, and two senior high schools) were randomly selected from all schools (24 primary schools, 27 junior high schools, and six senior high schools) in Kaiyuan; in the second stage, according to the required sample size, 4–6 classes were randomly selected from each of the chosen schools.

For all students within the chosen classes, only individuals aged above 10 years and below 18 years were preliminarily included as eligible subjects. To set a lower age limit of 10 years is because, in this study, we simultaneously measured suicidal ideation and behaviors, only children aged 10 years and above can fully understand the definition and consequence of suicide (10). Subjects were further excluded by using the following criteria: (1) illiteracy; (2) serious mental diseases (clinically diagnosed psychosis, schizophrenia, substance-induced mood disorders, neurosis, and personality disorder) or physical diseases; (3) difficulties in hearing or communicating; and (4) refuse to participate.

After obtaining the signed informed consent from the legal guardians of the eligible subjects, a self-administered questionnaire survey was conducted. Considering that a self-administered survey is prone to miss data, after the completion, every questionnaire was carefully checked and reviewed by pre-trained quality control personnel, who were master's students either in psychiatry or in public health from Kunming Medical University, or local health professionals recruited in Kaiyuan.

In this study, we only included study participants with depressive symptoms, screened by using the Patient Health Questionnaire-9 (PHQ-9), with a recommended cutoff value of 4 (11). The study protocol was reviewed and approved by the Ethics Review Committee of Kunming Medical University.

### Measurements

The questionnaire we used is a comprehensive instrument containing multiple modules. In this study, our analysis was based on the following parts: general characteristics (e.g., demographics, socioeconomic indicators, and family features), SH behaviors, hypomanic symptoms, and depressive symptoms.

#### SH behaviors

The definition of SH used in this study indicates the act with the purpose of harming oneself, regardless of intention (1). SH behaviors were measured using the Modified version of Adolescents Self-Harm Scale (MASHS) developed by Feng (12), adapted from the Deliberate Self-Harm Inventory (DSHI) (13). The validity and reliability of MASHS had been validated in the Chinese adolescent population. The scale measures the lifetime prevalence of the 18 most commonly observed SH behaviors in the Chinese adolescents. Likert-style responses are used to assess frequency (never, once, two to four times, five times, and above) and severity (non-observable injury, slight injury that needs no treatment, medium injury that requires simple medical treatment, severe injury that requires treatment in medical facilities, and critical injury that requires urgent treatment in emergency room) of SH. The Cronbach's α of MASHS for this study sample is 0.882 (bootstrap 95% CI: 0.863–0.897). Repeated SH was defined as two or more times of SH, and severe SH was defined as SH of medium and above severity.

#### Hypomania

Hypomanic symptoms of the respondents in the past 2 weeks were measured by using the 32-item Hypomania Checklist (HCL-32) (14). The validity of HCL-32 has been verified previously in the Chinese populations (15, 16), however, not in children or adolescents. The instrument consists of 32 questions. For each question, if the answer is confirmative, a score of 1 will be assigned; if the answer is negative, a score of 0 will be assigned. Respondents with a combined score of no <14 were considered hypomanic (14). Previous studies have suggested that the 32 items in the HCL-32 can be perfectly interpreted by two factors, which measure “active/elated” (factor 1, items 2, 3, 4, 5, 10, 11, 12, 13, 15, 16, 19, 20, 22, 24, and 28) and “risk-taking/irritable” (factor 2, items 7, 8, 21, 23, 25, 26, and 27) (15, 16). We further used quartiles of the combined scores of the two hypomanic factors to regroup subjects and analyzed their associations with SH prevalence, SH severity, and SH repetition. The Cronbach's α of HCL-32 for this study sample is 0.793 (bootstrap 95% CI: 0.777–0.808).

#### Depressive symptoms

The PHQ-9 was used to measure depressive symptoms (17). This instrument has nine items, and each item has four possible options in frequencies of the described situations in the past 2 weeks (not at all, only a few days, more than half of the days, and almost every day), with an assigned score from 0 to 3, and the combined score for PHQ-9 ranges between 0 and 27 points. Respondents with a PHQ-9 score of higher than four points were deemed positive (11). Published studies suggested that for adolescent populations, sensitivity and specificity of the PHQ-9 are similar to those of adult populations (18). The Cronbach's α of PHQ-9 for this study sample is 0.746 (bootstrap 95% CI: 0.717–0.773).

### Statistical analysis

Descriptive statistics were calculated to describe the general characteristics of the participants. A univariate logistic regression model was used to screen for possible related factors of SH. A multivariate binary logistic regression model was then used to estimate the adjusted association between hypomanic symptoms and SH. Two multivariate logistic regression models were fitted sequentially: model 1 focused on the association between hypomanic symptoms and SH, and model 2 estimated the associations between the two hypomanic factors and SH. Subgroup analysis was performed exclusively for SH subjects to further estimate the associations between hypomanic symptoms (and the two factors) and repetition and severity of SH behaviors.

All data were analyzed by using the R software (version 3.6.2, The R Foundation for Statistical Computing, Vienna, Austria). Survey data-related packages were used to adjust for possible inter-correlation between subjects sampled from the same cluster (the same class from the same school). The statistical significance level was set as *p* < 0.05, two-tailed, only except for univariate logistic regression, which adopted a comparatively loose criterion of *p* < 0.10, as suggested previously (19). Study subjects with missing information in analytical variables were deleted.

## Results

### General information

Initially, 5,132 eligible students were contacted, and 4,858 students were successfully surveyed, with a response rate of 94.66%. Among the 4,858 respondents, 249 respondents were excluded due to incomplete information. There were altogether 1,577 students who screened positive for depressive symptoms by using the PHQ-9 at a cut-off value of 4; all of them were included in the final analysis.

The major features of the 1,577 study subjects are displayed in Table 1. Among them, 1,008 reported SH behavior, with an estimated prevalence of 63.92% (95% CI: 58.70–69.00%). For SH adolescents, 60.70% (95% CI: 65.10–74.00%) and 37.50% (95% CI: 30.70–45.00%) had experienced repeated and severe SH. A total of 646 (40.96%, 95% CI: 33.00–49.00%) adolescents reported hypomanic symptoms based on a criterion of HCL-32≥14, and the median for HCL-32 scores was 12, with an interquartile range (IQR) of 8. The medians (IQRs) for factor I and factor II of HCL-32 were 7 (6) and 3 (2), respectively.

**Table 1:** General features of the study subjects, Kaiyuan, Yunnan, 2020 (*N* = 1,577).

**Features**	***N*** **(%)**	**Mean (SE)^§^/Median (IQR)^¶^**
Sex: boys	636 (40.33)	
Age		14.10 (0.37)^§^
Ethnicity		
Han majority	459 (29.11)	
Other minorities	1118 (70.89)	
**Grade**		
Primary school	298 (18.90)	
Junior high school	978 (62.02)	
Senior high school	301 (19.08)	
Single child in the family: No	1189 (75.40)	
Father's age		42.05 (0.43)^§^
Mother's age		39.65 (0.47)^§^
**Father's education level**		
Primary school and below	554 (35.13)	
Junior high school	504 (31.96)	
Senior high school and above	348 (22.07)	
Missing or unknown	171 (10.84)	
**Mother's education level**		
Primary school and below	632(40.08)	
Junior high school	422 (26.76)	
Senior high school and above	325 (20.61)	
Missing or unknown	198 (12.55)	
**Father's health status**		
Any diagnosed illness	719 (45.59)	
No diagnosed illness	858 (54.41)	
**Mother's health status**		
Any diagnosed illness	668 (42.36)	
No diagnosed illness	909 (57.64)	
**Parents' marital status**		
In marriage	1313 (83.26)	
Not in marriage	264 (16.74)	
HCL-32 combined score		12 (8)^¶^
HCL-32 factor 1 score		7 (6)^¶^
HCL-32 factor 2 score		3 (2)^¶^
Hypomanic symptoms: Yes (HCL-32≥14)	646 (40.96)	
SH behavior: Yes	1008 (63.92)	

### Hypomanic symptoms with SH prevalence

With SH prevalence as the dependent variable, Table 2 presents the results of univariate and multivariate logistic regression models. At a lower significance level of *p* < 0.10, sex, grade, father's education level, mother's education level, parents' marital status, and hypomanic symptoms were included in the subsequent multivariate logistic regression models. Although in multivariate model 1, after adjusting for covariates, the presence of hypomanic symptoms, in general, was not statistically associated with SH (adjusted odds ratio [OR]: 0.75, 95% CI: 0.54–1.05), in multivariate model 2, when the two hypomanic factors were simultaneously analyzed, both of them presented as significantly associated factors of SH: every 1-point increase in factor I and factor II scores was associated with ORs of 0.94 (95% CI: 0.91–0.97) and 1.25 (95% CI: 1.15–1.36). In comparison, we also evaluated the associations between hypomanic symptoms and SH for children and adolescents without depression, and the fitting results were comparable (refer to Supplementary Table S1).

**Table 2:** Univariate and multivariable logistic regression models fitting results for SH.

**Covariates**	**Univariate model**	**Multivariate model 1**	**Multivariate model 2**
	**Crude OR (90% CI)**	**Adjusted OR (95% CI)**	**Adjusted OR (95% CI)**
Sex: Girls (Ref: Boys)	1.57 (1.34, 1.83)	1.53 (1.22, 1.90)	1.53 (1.21, 1.93)
Age: +1 year	0.98 (0.88, 1.09)		
Ethnicity: Other minorities (Ref: Han)	0.89 (0.71, 1.11)		
**Grade (Ref: Primary school)**			
Junior high school	1.62 (1.20, 2.17)	1.38 (0.89, 2.14)	1.37 (0.86, 2.17)
Senior high school	0.93 (0.62, 1.39)	0.80 (0.46, 1.40)	0.86 (0.52, 1.43)
Single Child: Yes (Ref: No)	0.96 (0.81, 1.14)		
Father's age: +1 year	0.99 (0.96, 1.01)		
Mother's age: +1 year	0.99 (0.97, 1.01)		
**Father's education level (Ref: Primary school and below)**			
Junior high school	1.30 (1.07, 1.58)	1.21 (0.85, 1.71)	1.24 (0.85, 1.80)
Senior high school and above	1.08 (0.77, 1.53)	0.89 (0.48, 1.63)	0.89 (0.48, 1.65)
**Mother's education level (Ref: Primary school and below)**			
Junior high school	1.31 (1.04, 1.64)	1.29 (0.90, 1.85)	1.34 (0.92, 1.93)
Senior high school and above	1.31 (1.01, 1.69)	1.57 (0.98, 2.53)	1.75 (1.09, 2.80)
Father's health status: Any illness (Ref: No illness)	1.20 (0.90, 1.61)		
Mother's health status: Any illness (Ref: No illness)	1.23 (0.90, 1.68)		
Parents' marital status: Not in marriage (Ref: In marriage)	1.42 (1.19, 1.71)	1.28 (1.01, 1.62)	1.27 (0.96, 1.67)
Hypomanic symptoms: Yes (Ref: No)	0.75 (0.57, 0.99)	0.75 (0.54, 1.05)	
Hypomanic factor I: +1 point			0.94 (0.91, 0.97)
Hypomanic factor II: +1 point			1.25 (1.15, 2.36)

**Figure 2:**
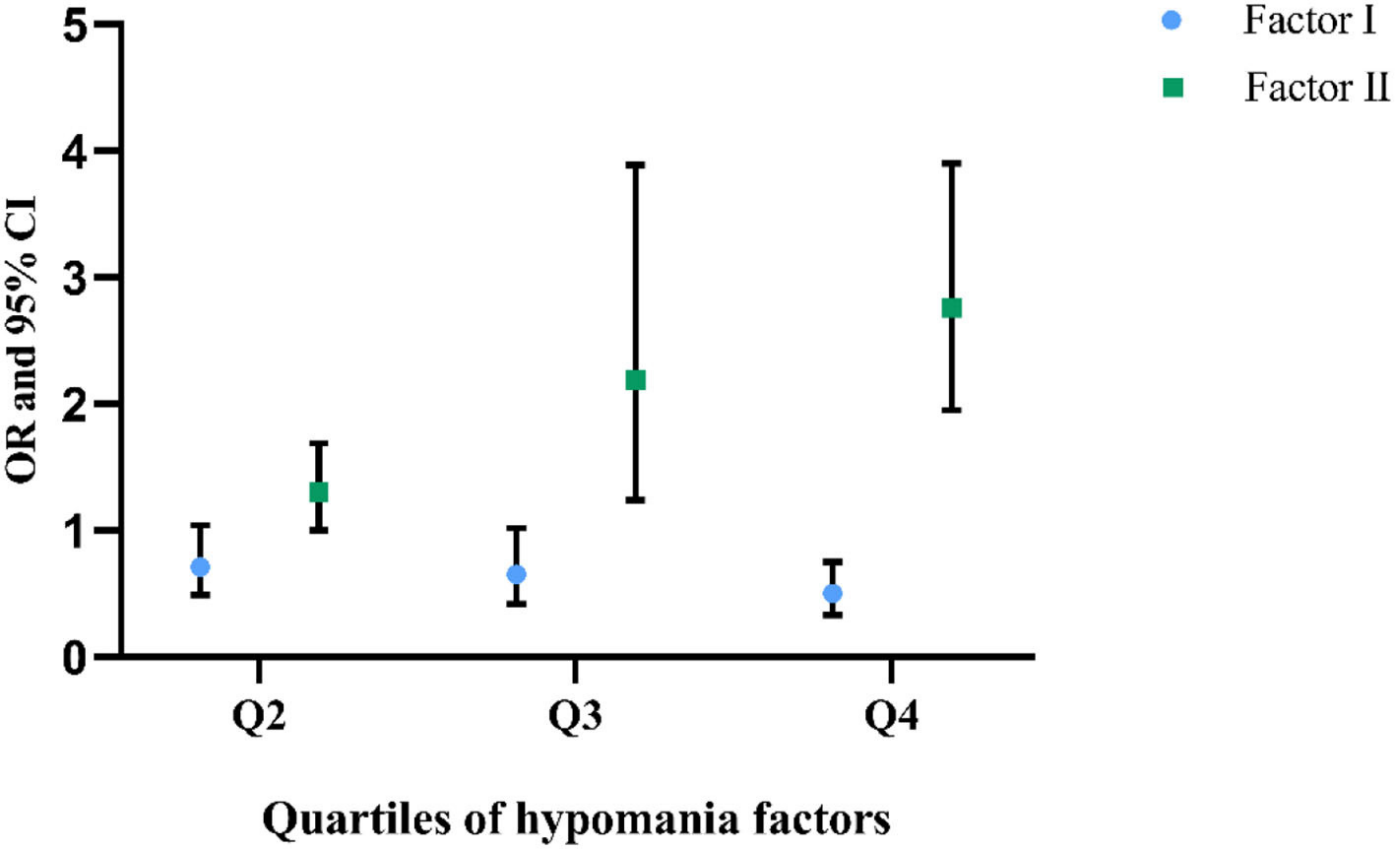
Quartiles of hypomania factors with SH prevalence (reference: Q1).

We further used quartiles of the two factors to divide the participants: Q1 (factor I < =4, factor II < =2), Q2 (4 < factor I < =7, 2 < factor II < =3), Q3 (7 < factor I < =10, 3 < factor II < =4), and Q4 (10 < factor I < =15, 4 < factor II < =7). When using Q1 as the reference group, a prominent dose–response association has been identified for the association between factor II and SH: along with the increase of factor II score, the risk of SH also increased; Q2, Q3, and Q4 were associated with adjusted ORs of 1.30 (95% CI: 1.00–1.69), 2.19 (95% CI: 1.24–3.89), and 2.76 (95% CI: 1.95–3.90), respectively. Nevertheless, for factor I, the dose–response association was less obvious; compared with Q1, only Q4 was associated with statistically decreased SH risk (adjusted OR: 0.50, 95% CI: 0.33–0.75) (Figure 2).

### Hypomanic symptoms with SH repetition and severity

**Figure 3:**
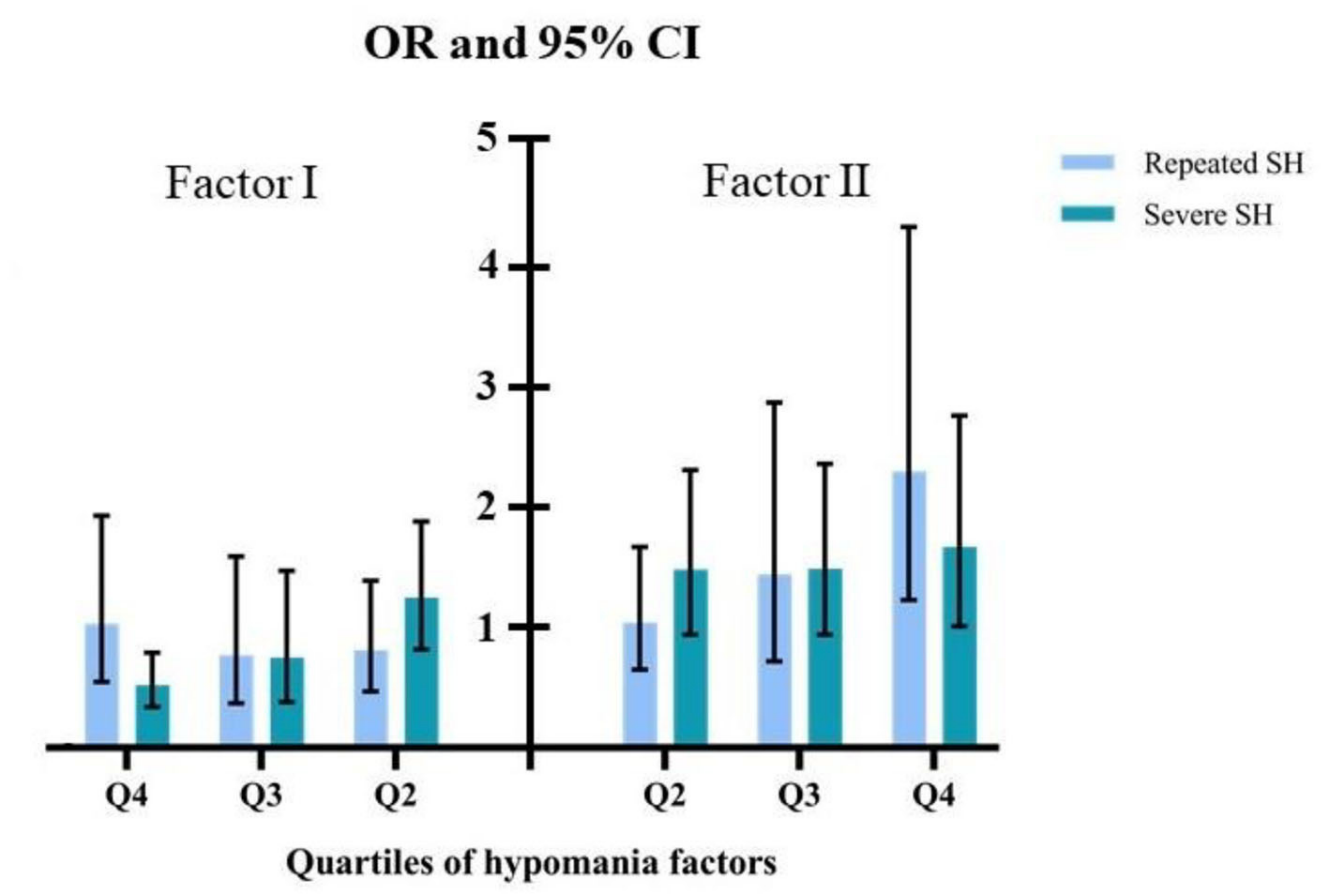
Quartiles of hypomania factors with SH severity and repetition (reference: Q1).

Among the 1,008 respondents reported SH behaviors, 703 (69.74%, 95% CI: 65.10–74.00%) had repeated SH, and 378 (37.50%, 95% CI: 30.70–45.00%) had SH of medium and above severity. We also used quartiles of the two hypomanic factors to investigate their associations with SH repetition and severity. For both repeated SH and severe SH, along with the increase of factor II score, an identifiable upward trend in the association has been revealed: compared to respondents with a Q1 factor II score, the adjusted ORs for SH repetition and severity were 1.31 (95% CI: 0.96–1.80) and 1.38 (95% CI: 0.81–2.33) for Q2, 1.37 (95% CI: 0.96–1.95) and 1.57 (95% CI: 0.95–2.61) for Q3, and 2.05 (95% CI: 1.47–2.80) and 1.87 (95% CI: 1.09–3.22) for Q4. For factor I, no obvious trend has been identified; compared with Q1, the adjusted ORs for SH repetition and severity were 0.81 (95% CI: 0.47–1.39) and 1.25 (95% CI: 0.82–1.88) for Q2, 0.77 (95% CI: 0.37–1.59) and 0.75 (95% CI: 0.38–1.47) for Q3, and 1.03 (95% CI: 0.55–1.93) and 0.52 (95% CI: 0.34–0.79) for Q4 (Figure 3).

## Discussion

In this study, we found a high prevalence (40.96%) of hypomanic symptoms in the Chinese children and adolescents with depression. However, a previous study has reported an even higher hypomania prevalence of 55–62% in patients with depression (20). Although the same instrument (HCL-20) had been used, different study populations may contribute to this discrepancy, as this study only included individuals with depressive symptoms other than clinically diagnosed depressive disorders. For the two major hypotheses, although the association between hypomania and SH cannot be supported after controlling for prominent covariates, the two hypomanic factors were significantly but discordantly associated with SH as expected: factor I was associated with decreased risk of SH, whereas factor II was associated with increased risk of SH.

Among the two hypomanic factors, factor I is the socially positive and advantageous facet, and it has been labeled as “active/elated” and mainly reflects hyperactivity, mood elation, and active thinking. Therefore, factor I is more closely related to the positive thinking of an individual, and it has been found that optimism can significantly ameliorate depression and stress and strengthen coping skills, all of which are protective factors of SH (17). On the contrary, factor II is the socially negative side of hypomania, which characterizes risk-taking behavior, anger, irritability, and avoidance of thoughts. It has been found that factor II is overly expressed among individuals with BD (14). BD has been associated with increased SH risk: a cross-sectional study showed that the prevalence of SH among adolescents with BD was 37%, significantly higher than adolescents without BD (13–23%) (7). Emotional dysregulation might be involved in connecting BD with SH in children and adolescents, as BD is centrally characterized by impaired emotion regulation (21), a recognized risk factor of SH in children and adolescents (22). Besides, factor II is also indicative of individual impulsivity (15). A published meta-analysis that synthesized 28 studies concluded that mood-based impulsivity-related traits were consistently associated with increased lifetime SH in adolescent populations (23).

Although both factors were significantly associated with SH, one interesting finding of this study is that the dose–response association was more apparent for factor II. Similarly, when further exploring the associations between hypomanic factors and characteristics of SH among self-harmed adolescents, only the increase of factor II score was related to an elevated risk of SH severity and SH repetition. Compared to one-off SH behavior, repeated SH and severe SH can substantially increase suicidal risk in adolescents. For instance, a multicenter study of children and adolescents in England showed that among SH individuals, subjects with repeated self-harming behaviors reported 2.7-fold suicidal risk longitudinally (24). All these results may suggest the discrepant SH risk related to hypomanic factors in teenagers with depression.

The results of this study may suggest that, compared with factor I, factor II is probably of greater importance in formulating SH intervention measures for depressive children and adolescents with hypomanic symptoms. As mentioned earlier, because factor II is intimately associated with impulsivity and irritability, therefore, intervention measures targeting these two characteristics could be considered. Some effective school-based intervention methods which aim at reducing impulsivity of children and adolescents from different perspectives have been reported: focusing on enhancing family-school communication, parental behavior management, or academic instruction skills (25). A newly published meta-analysis by Vekety et al. (26) revealed that mindfulness-based interventions, which are easily implemented in educational practice, were generally effective in reducing impulsivity. However, as the experimental evidence of these intervention measures remains scarce in the Chinese adolescent populations, their effectiveness needs to be corroborated further.

For treating irritability, some medications were found effective in children and adolescents, such as antipsychotic drugs, stimulants, and selective serotonin reuptake inhibitors (SSRIs) (27). However, for SSRIs, only indirect evidence is currently available, as published randomized controlled trials (RCTs) that support its effectiveness in reducing irritability were mostly based on adults; hence, research is desperately needed in youth (28). Even if it is effective in dealing with irritability for children and adolescents, the use of SSRIs should be prudent, considering the possibility of induced mania (29). Aside from psychopharmacological agents, psychotherapeutic approaches such as parent training and cognitive behavioral therapy (CBT) were also important for reducing pediatric impulsivity (30).

Although this study is among the first attempt to discuss the independent association between hypomania and SH among the Chinese children and adolescents with depression, an important issue lacks due investigation. Some limitations, such as the inability in causal inference for cross-sectional design, possible information bias caused by the self-reporting method, and limited representativeness of the study sample to the general Chinese children and adolescent population, should be noticed when interpreting the major findings. Besides, considering the difficulty with the hypomania concept and measurement, in general, in particular, when using survey methods in a non-clinical population, although we tried to improve the accuracy in measuring hypomania by only including individuals with depressive symptoms, the chance of misclassification may exist. Finally, we analyzed individuals with depressive symptoms, which are different from clinically diagnosed depressive disorders; therefore, the major findings of this study could be inapplicable to patients with depression. For future studies, considering that higher suicidality in hypomania could be associated with a higher level of impulsivity or higher severity of mood symptoms, which mechanism plays a more essential role in the presence of SH should be further investigated.

## Conclusion

In this population-based cross-sectional study, we found that the two hypomanic factors were independently and discordantly associated with SH among a large sample of the Chinese children and adolescents with depressive symptoms. Besides, among the two hypomanic factors, a more prominent dose–response association has been revealed between factor II and SH. The major findings of this study suggest that, for hypomanic children and adolescents, individuals with higher factor II score could be at further increased SH risk; therefore, they should be prioritized for intervention.

## Data availability statement

The raw data supporting the conclusions of this article will be made available by the authors, without undue reservation.

## Ethics statement

The studies involving human participants were reviewed and approved by the Ethics Review Board of Kunming Medical University. Written informed consent to participate in this study was provided by the participants' legal guardian/next of kin.

## Author contributions

YX and CX designed the study. YC, HR, AD, SW, JP, and LC carried out the data collection. DF and YC performed data analysis. DF prepared the draft manuscript. YX critically revised the manuscript. All authors critically revised the manuscript for important intellectual content.

## Funding

This study was supported by the National Natural Science Foundation of China [Grant No. 82060601], the Top Young Talents of Yunnan Ten Thousand Talents Plan [Grant No. YNWR-QNBJ-2018-286], and the Innovative Research Team of Yunnan Province [Grant No. 202005AE160002].

## Conflict of interest

The authors declare that the research was conducted in the absence of any commercial or financial relationships that could be construed as a potential conflict of interest.

## Publisher's note

All claims expressed in this article are solely those of the authors and do not necessarily represent those of their affiliated organizations, or those of the publisher, the editors and the reviewers. Any product that may be evaluated in this article, or claim that may be made by its manufacturer, is not guaranteed or endorsed by the publisher.
