# Supplementary material for: Association between hypomania and self-harm behaviors in Chinese children and adolescents with depressive symptoms
Source: Front Psychiatry. 2022 Aug 24;13:870290. doi: 10.3389/fpsyt.2022.870290 (PMC9449148; doi:10.3389/fpsyt.2022.870290)
Supplement: Supplementary file 1 [file Table_1.pdf]

Table S1 Univariate and multivariable Logistic regression models fitting results for SH in non-depressive children and adolescents (*N*=3032).

Covariates	Univariate model<br>Crude OR (90% CI)	Multivariate model 1<br>Adjusted OR (95% CI)	Multivariate model 2<br>Adjusted OR (95% CI)
Sex: Girls (Ref: Boys)	0.83 (0.68, 1.02)		
Age: +1 year	1.23 (1.17, 1.29)	1.06 (0.93, 1.21)	1.05 (0.92, 1.20)
Ethnicity: Other minorities (Ref: Han)	1.08 (0.82, 1.43)		
Grade (Ref: Primary school)			
Junior high school	2.42 (1.84, 3.20)	1.82 (0.99, 3.35)	1.76 (0.99, 3.12)
Senior high school	2.44 (1.97, 3.02)	1.61 (0.74, 3.51)	1.69 (0.79, 3.59)
Single Child: Yes (Ref: No)	0.77 (0.58, 1.02)		
Father's age: +1 year	0.96 (0.89, 1.04)		
Mother's age: +1 year	1.00 (0.92, 1.09)		
Father's education level (Ref: Primary school and below)			
Junior high school	0.76 (0.61, 0.95)	0.82 (0.64, 1.06)	0.86 (0.66, 1.11)
Senior high school and above	0.58 (0.42, 0.79)	0.77 (0.53, 1.12)	0.80 (0.55, 1.16)
Mother's education level (Ref: Primary school and below)			
Junior high school	0.76 (0.59, 0.98)	0.87 (0.63, 1.06)	0.90 (0.65, 1.24)
Senior high school and above	0.65 (0.47, 0.89)	0.94 (0.60, 2.49)	1.05 (0.68, 1.61)
Father's health status: Any illness (Ref: No illness)	1.71 (1.33, 2.19)	1.17 (0.76, 1.80)	1.15 (0.72, 1.81)
Mother's health status: Any illness (Ref: No illness)	1.81 (1.35, 2.43)	1.17 (0.78, 1.75)	1.10 (0.73, 1.66)
Parents' marital status: Not in marriage (Ref: In marriage)	1.24 (1.02, 1.50)	1.35 (0.94, 1.92)	1.28 (0.88, 1.87)
Hypomanic symptoms: Yes (Ref: No)	1.28 (1.05, 1.55)	1.09 (0.88, 1.34)	
Hypomanic factor I: +1 point			0.97 (0.94, 1.01)
Hypomanic factor II: +1 point			1.32 (1.20, 1.46)
